# Non-traumatic Chronic Subdural Hematoma With Myelodysplastic Syndrome: A Case Report

**DOI:** 10.7759/cureus.36110

**Published:** 2023-03-14

**Authors:** Muhammad Irfan, Sowjanya Kurakula, Harmandeep Singh, Karanbir Singh

**Affiliations:** 1 Department of Neurosurgery, Nishtar Medical University, Multan, PAK; 2 Department of Obstetrics and Gynecology, Mamata Academy of Medical Sciences, Khammam, IND; 3 Department of Internal Medicine, Government Medical College, Amritsar, IND

**Keywords:** outcome, rare association, chronic subdural hematoma, myelodysplastic syndrome, case report

## Abstract

Spontaneous chronic subdural hematoma is a rare condition that appears in association with myelodysplastic syndrome. A 25-year-old male with a known case of myelodysplastic syndrome presented to the emergency department with a headache and loss of consciousness. Considering ongoing chemotherapy, burr hole trephination of the chronic subdural hematoma was performed, and he was discharged following a successful procedure. To the best of our knowledge, this is the first report of myelodysplastic syndrome with spontaneous chronic subdural hematoma.

## Introduction

Chronic subdural hematoma is commonly caused by trauma due to the cortical bridge veins or fragile vessels that run between the dura and surface of the membrane and multiple microhemorrhages [1]. A spontaneous chronic subdural hematoma can be associated with neoplasms, coagulation disorders, or rupture of cerebral aneurysms and arteriovenous fistula, cocaine, and arachnoid cyst. Here we report a case of a non-traumatic chronic subdural hematoma associated with myelodysplastic syndrome, a hematological disorder characterized by abnormal formation of blood cells in the bone marrow, which can lead to either fatal cytopenias or acute myelogenous leukemia. Chronic subdural hemorrhage (SDH) is usually more common in elderly patients after any traumatic event and is associated with a high morbidity and mortality rate. But cases with chronic subdural hematoma associated with myelodysplastic syndrome are very few. Our case illustrates the association of chronic subdural with myelodysplastic syndrome and its management.

## Case presentation

We present a case of a 25-year-old male presented with complaints of generalized body weakness, bone pains, and low-grade fever for two years with worsening exertional fatigue, palpitation, and shortness of breath for two weeks. His complete blood count showed the hemoglobin level at 9.3 g/dL, platelet count 37 x 10^9^/L, WBC count 2.58 x 10^9^/L, neutrophils 25%, and lymphocytes 46%. The bone marrow aspiration report showed an absolute reticulocyte count of 2.5 x 10^12^/L and pancytopenia with severe neutropenia, dysplastic neutrophils, occasional myelocytes, metamyelocytes and 2% atypical mononuclear cells; however, bone marrow trephination revealed single core composed of subcortical and medullary marrow exhibiting prominent histiocytes, loss of marrow adipose tissue and hypercellularity. Overall, cellularity was around 85%. Immunohistochemistry of CD34 markers highlighted blast cells, and CD41 and CD61 markers highlighted dysplastic megakaryocytes. Abdomen ultrasound revealed hepatosplenomegaly. Other laboratory and imaging findings were insignificant, and he was diagnosed with myelodysplastic syndrome; therefore, chemotherapy protocols such as depot injection of azacitidine and venetoclax orally were commenced by the hemato-oncology team.

**Figure 1:**
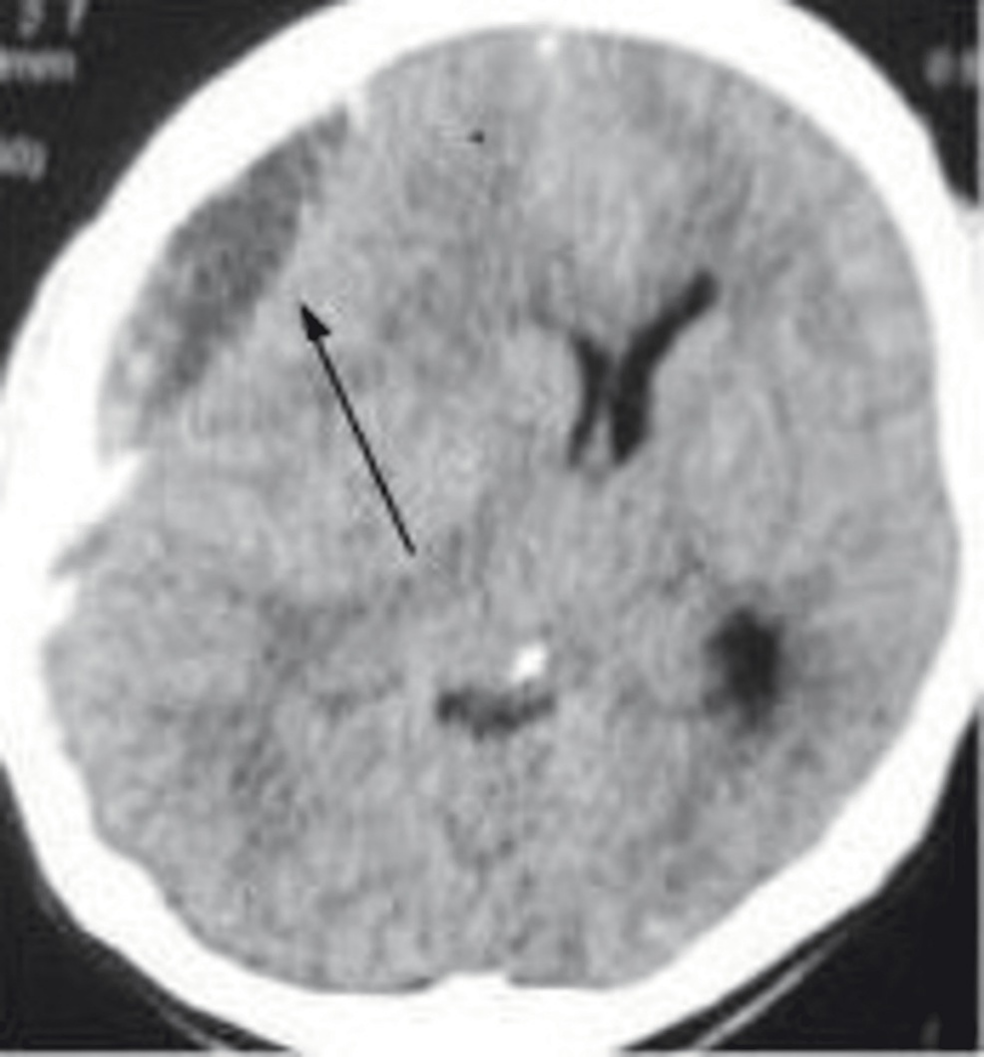
A CT scan of the brain with axial view showing a mixed density picture of the hyperdense and hypodensce crescent-shaped lesion in the right fronto-parietal region with a midline shift (arrow)

**Figure 2:**
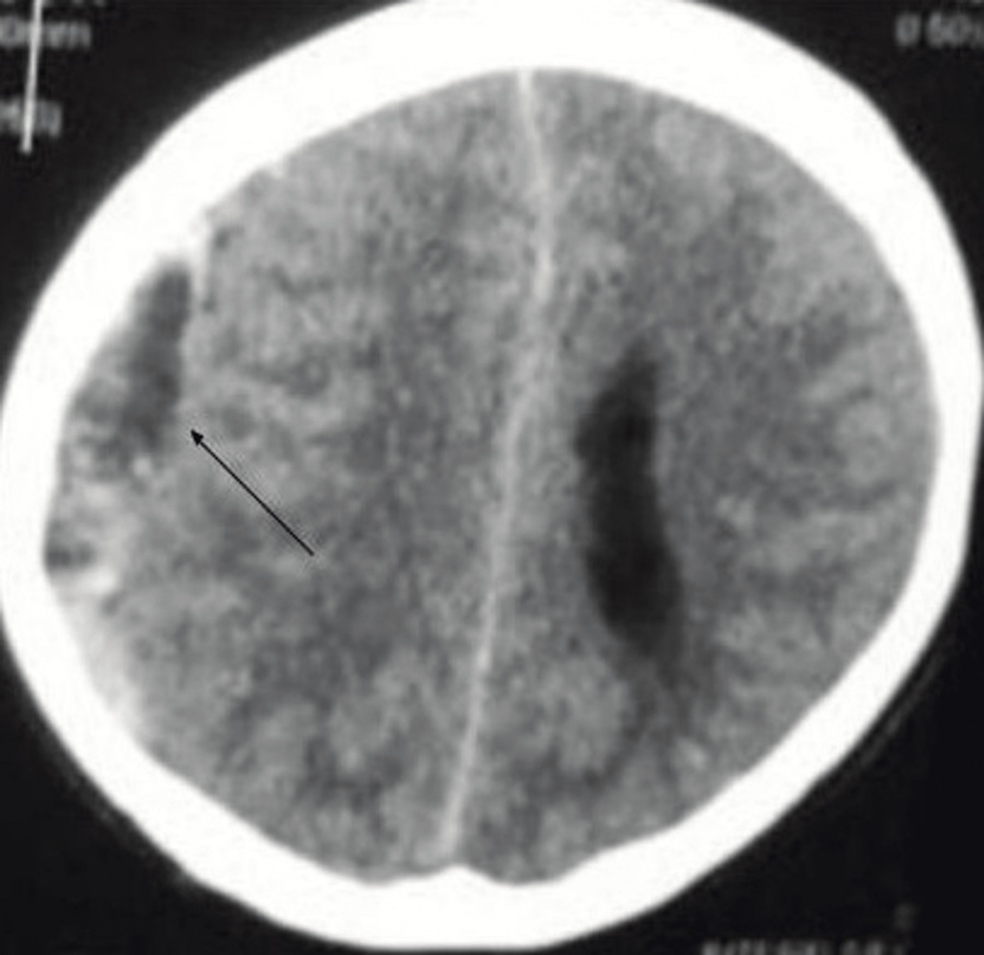
A CT scan of the brain with axial view showing a hypodense crescent-shaped lesion in the fronto-parietal region (arrow)

After five months, he was admitted to the emergency department with complaints of altered levels of consciousness for three days, headache, and vomiting for one day. He was alert and conscious. When his vital signs were checked, his temperature was 98.6°F, pulse rate was 90 beats per minute, respiratory rate was 18 per minute, blood pressure was 120/70 mm Hg, and BMI was 26. His neurological examination revealed no sign of limb weakness, dysarthria, or ataxic gait, and sensory and cranial examination findings were normal. However, his pupils were mildly reactive, and plantar reflexes were bilaterally upgoing. His Glasgow Coma Scale (GCS) score was 14/15. However, other systemic examination findings were insignificant. An initial non-contrast CT scan of the brain window with axial view showed a crescent-shaped lesion with mixed density picture of hyperdensity and hypodensity on the right frontal-temporal-parietal region causing a 10-mm midline shift towards the left side along with lateral ventricular effacement (as shown in Figures 1, 2).

**Figure 3:**
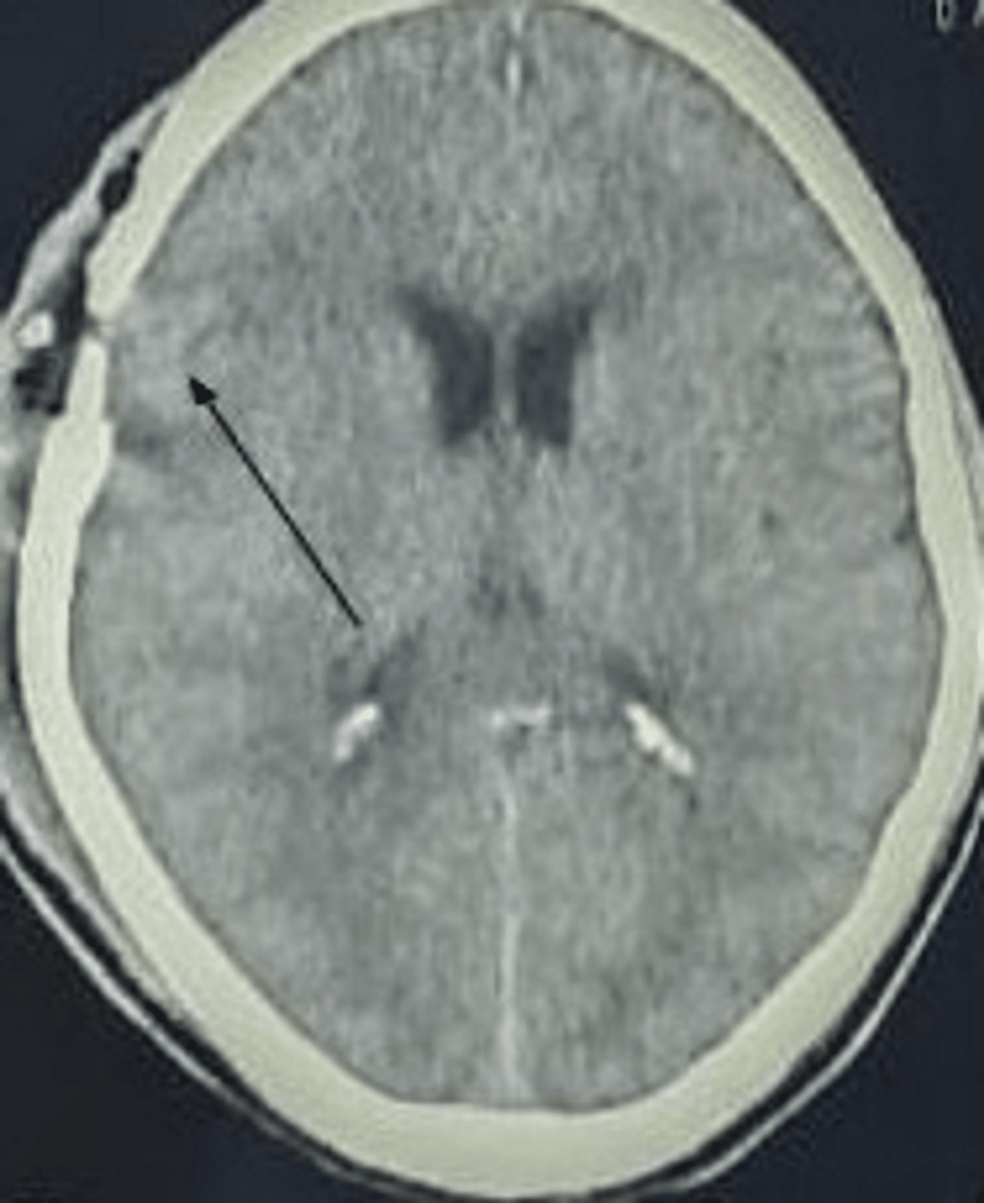
A post-operative CT scan of the brain with axial view showing evacuation of the hematoma after burr hole trephination of the chronic subdural hematoma (arrow)

A serum blood test showed an RBC count of 2.58 x 10^3^/uL, hemoglobin level at 6.3 g/dL, mean corpuscular volume 71.6 fL, hematocrit 16.4%, platelet count 20 x 10^3^/uL, WBC count 2.2 x 10^6^/uL, neutrophils 25%, lymphocytes 70%, monocytes 3%, and eosinophils 2%. The liver function test and serum electrolytes were within normal limits, and the random blood sugar level was 145 mg/dL. Intravenous fluid was given, and packed RBC and platelets were transfused, which enhanced the hemoglobin level from 6.3 to 7.4 g/dL and platelet level from 20 x 10^3^/uL to 35 x 10^3^/uL. In addition, he was administered with mannitol and levetiracetam intravenously. After taking informed consent, the patient was prepared for surgery on the second day of admission. Considering the recommended surgical protocol, a burr hole trephination procedure was performed on the right fronto-parietal region, 60 mL of blood was aspirated, and the patient was kept under observation in the recovery room for the next 24 hours. On the next day, a post-operative CT scan of the brain was done (Figure 3).

After the fourth post-operative day in the neurosurgery ward, the patient was discharged and kept on follow-up in the outpatient unit.

## Discussion

Traditionally, a chronic subdural hematoma is usually a result of any traumatic event that causes the tearing of bridging veins traversing from the brain to the draining dural-venous sinuses, consequently leading to accumulation of blood in the subdural space over time that ultimately becomes symptomatic after four to seven weeks associated with several ongoing processes such as angiogenesis, fibrinolysis, and inflammation [2]. A chronic subdural hematoma, conventionally a part of traumatic brain injury, is associated with older individuals, athletes who play contact sports, patients on anticoagulants, those with inherited bleeding disorders like hemophilia, alcohol use disorder, and abusive head trauma in children. Each year, 1.72-20.6 cases of chronic subdural hematomas per 100,000 persons are reported [3]. The estimated incidence of subdural hemorrhage is expected to reach 17.4 per 100,000 individuals by 2030, along with projected annual cases of 60,000 patients with chronic subdural hemorrhage [4]. There are many risk factors for chronic subdural hematomas such as old age, male gender, and antiplatelet and anticoagulant use. A spontaneous chronic subdural hematoma due to a tumor appears most commonly because of solid tumor metastasis. Sometimes, chronic subdural hematomas can occur due to hematological malignancies [5]. A case of chronic subdural hematoma caused by acute myeloblastic leukemia in a 67-year-old male has been reported [6].

The pathophysiology of a chronic subdural hematoma due to hematological tumors is still under study. But some researchers have suggested that dural metastasis from any leukemia could disrupt dural venous sinuses and accumulation of blood under the dura mater leading to chronic subdural hematomas in patients with leukemia [7]. Our case discussed here is associated with myelodysplastic syndrome, a type of blood cancer that affects the bone marrow and production of blood cells and which is characterized by low blood count, low platelets, and a high risk of developing acute myeloid leukemia. A total of 15% of cases of myelodysplastic syndrome occur after chemotherapy or radiotherapy for previous cancer; the elderly population is most commonly affected by the myelodysplastic syndrome [8]. A few cases of myelodysplastic syndrome associated with chronic subdural hematoma have been reported in the existing literature.

Non-traumatic chronic subdural hematoma could also have appeared with some other rare conditions such as arachnoid cysts, intracranial meningioma, AL amyloidosis (amyloid light chain or primary amyloidosis), rupture of cerebral aneurysm, arteriovenous fistula and cocaine use [9-11]. Chronic subdural hematoma, irrespective of any predisposing risk factors, is usually managed by the surgical procedure termed burr hole trephination; however, decompressive craniectomy is done in the case of acute subdural hematoma. The prognosis of acute subdural hematoma is poor compared to that of chronic subdural hematoma. As seen in a study, the survival rate of patients is 80.3% for chronic subdural hematoma and 69.7% for acute subdural hematoma after undergoing relevant surgical procedures [12].

A study has also shown that the outcomes in non-traumatic subdural hemorrhage are worse in patients with coagulation deficiency compared to patients with hematomas of arterial origin without coagulation deficiency [13]. Research was done on patients with hematologic malignancies with concurrent subdural hematoma with surgical and non-surgical treatments, and the outcome was assessed. The study concluded that the variables like advanced age, low hemoglobin, low platelet counts, and surgical intervention are associated with increased mortality [4].

Therefore, because of this existing evidence for the poor outcome of non-traumatic chronic subdural hematoma due to any risk factor, it is being emphasized, for physicians and neurosurgeons, that underlying risk factors should be figured out and addressed immediately for better prognosis of non-traumatic chronic subdural hematoma. Our case, presented here, is also one of the examples of a non-traumatic chronic subdural hematoma linked to a myelodysplastic syndrome, which is a unique finding in our observation. So by reporting this case, we emphasize the consideration of myelodysplastic syndrome, one of the underlying risk factors for non-traumatic chronic subdural hematoma, and highlight the outcome of early management of chronic subdural hematomas associated with myelodysplastic syndromes; further future research on this should be encouraged.

## Conclusions

We conclude that chronic subdural hematoma could be a rare complication of myelodysplastic syndrome that may lead to a severe neurological deficit. Therefore, its early identification and management have a crucial role in the prognosis of associated chronic subdural hematoma.
